# Atypical Presentation of Herpes Simplex Virus Infection in an Immunocompromised Patient

**DOI:** 10.7759/cureus.37465

**Published:** 2023-04-12

**Authors:** Nada Alghamdi, Abdulelah Albaqami, Abdulmajeed Alharbi

**Affiliations:** 1 Department of Dermatology, King Fahad University Hospital, Dammam, SAU; 2 Medicine, Imam Abdulrahman Bin Faisal University, Dammam, SAU

**Keywords:** immunocompromised, atypical presentation, immunosuppression, hsv, herpes simplex infection

## Abstract

Immunocompromised patients are at risk of developing atypical herpes simplex virus (HSV) infection, which can be easily misdiagnosed. We present a case of a 69-year-old female who was receiving methotrexate and tofacitinib for a known case of rheumatoid arthritis. She was admitted to the ICU under neurology care after presenting with status epilepticus secondary to bacterial meningitis. She complained of a group of vesicles on the erythematous base accompanied by a burning sensation, erosions with a hemorrhagic crust that extended onto the vermilion lip, and painful oral mucosa erosion that involve the buccal, palatine, and tongue. The clinical differential diagnosis was herpes simplex infection, pemphigus vulgaris, paraneoplastic pemphigus, early drug-induced Stevens-Johnson syndrome, erythema multiform major, and methotrexate-induced mucositis. As the presentation was atypical, steroid treatment was initiated. Subsequent histopathology showed infectious dermatitis consistent with herpes virus infection. After discontinuing steroid treatment and starting an antiviral drug, the patient’s symptoms improved within a week.

There has been heightened clinical awareness about the atypical clinical presentation of herpes simplex infection in immunocompromised patients. HSV infection should be included in the differential diagnosis along with other vesiculobullous diseases.

## Introduction

Infection with herpes simplex virus (HSV), a double-stranded DNA virus, causes orofacial (mainly HSV-1) or genital (mainly HSV-2) herpes [1]. Transmission of both HSV-1 and HSV-2 occurs through direct contact and subsequently results in a lifelong infection. Most humans acquire HSV-1 early in life through the orolabial mucosa, while HSV-2 infections occur later through sexual transmission. Infection with one HSV type can typically stimulate immunity to prevent re-infection with the same but not with the other serotype [2]. It is estimated that 3.7 billion people below 50 years of age have been infected with HSV-1 [3].

As HSV requires intact cellular immunity to be controlled, immunocompromised patients are at risk of increased reactivation of HSV. However, even with immunocompetent humoral and cellular immune responses, HSV avoids eradication through several mechanisms of immune evasion. Multiple studies have shown active surveillance by T-cells both in the mucosa and in the trigeminal ganglia [4,5]. In immunocompromised patients, chronic mucocutaneous HSV infection may extend into deeper cutaneous layers, subsequently resulting in tissue necrosis. This can be accompanied by severe pain and atypical lesions upon clinical examination [6].

Patients with impaired cellular immunity are more susceptible to developing acyclovir-resistant infections compared with immunocompetent individuals. Persistence of HSV-1 ulcers despite multiple days of antiviral drugs should increase suspicion of acyclovir resistance [7]. In primary infection with HSV-1, clinical symptoms typically appear 2-12 days after the exposure, and may be characterized by the appearance of many lesions that are associated with pain (herpetic gingivostomatitis) and local enlargement of the lymph node [8,9]. Systemic symptoms include fever, malaise, and headache. If there is a delay in antiviral drugs, the lesions of primary HSV-1 infection may last for 12 days (ranging from 7 to 18 days) [10,11].

## Case presentation

**Figure 1:**
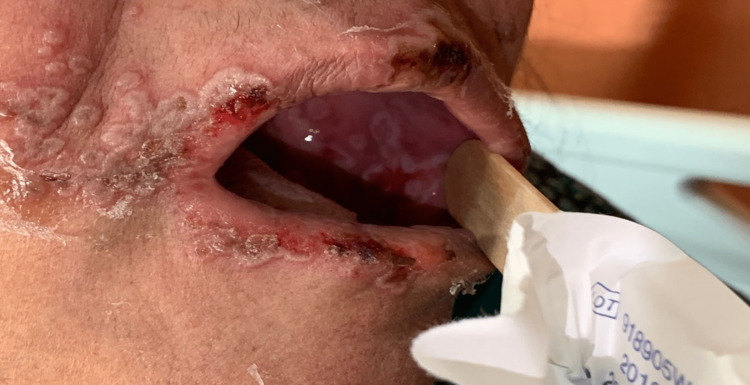
Erosions with hemorrhagic crusts on the lips and erosions on the buccal mucosa

**Figure 2:**
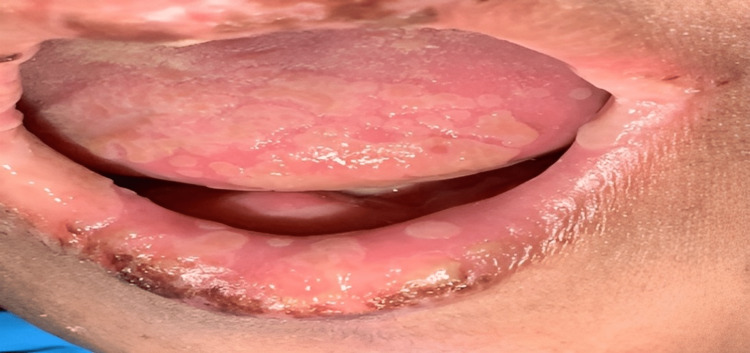
Erosions on the tongue and lips

A 69-year-old female with a known past medical history of rheumatoid arthritis, currently receiving methotrexate 15 mg PO weekly and tofacitinib 10 mg PO once daily. The patient was admitted to the ICU under the care of neurology as a case of status epileptics secondary to bacterial meningitis. Three days after the admission, she started complaining of a group of vesicles on the erythematous base accompanied by a burning sensation, erosions with a hemorrhagic crust on the lips, and painful oral mucosa erosion that involved buccal mucosa, palatine, and tongue. It was associated with difficulty swallowing solid and liquid food. The patient did not report skin lesions or the involvement of other mucosal sites. A review of her family history was non-contributory. Clinically, the patient appeared unwell and presented with a group of vesicles on the erythematous base accompanied by a burning sensation, erosions associated with hemorrhagic crusts that extended onto the vermilion lip, as well as painful multiple erosions of the oral mucosa that involved buccal mucosa, palatine, and tongue (Figures 1, 2).

The clinical differential diagnosis was herpes simplex infection, pemphigus vulgaris, paraneoplastic pemphigus, early drug-induced Stevens-Johnson syndrome, erythema multiform major, and methotrexate-induced mucositis. After three days, histopathology showed infectious dermatitis consistent with herpes virus infection. Oral and topical steroid treatment was discontinued, and oral acyclovir 400 mg three times a day was initiated for three weeks. The skin lesions and patient's symptoms improved within a week, and the lesions had a complete resolution by the end of the third week.

## Discussion

Atypical presentations of HSV infections have been documented during pregnancy, and in neonates, and are associated with the use of oral corticosteroids and immunosuppression after renal or bone marrow transplantation [12]. These infections typically involve the reactivation of a previously acquired HSV infection [13]. HSV infection can have many different presentations depending on the site of infection, the immune state of the patient, and whether the symptoms result from primary infection or recurrent infection. HSV infections can involve the skin, eye, oral mucosa, and genital area. Symptoms of HSV Infections tend to be unilateral, mild, and self-limiting, except in immunocompromised patients and newborns [14,15]. Clinical manifestations of atypical HSV infection in immunocompromised patients include large lesions, deep ulcers, satellite lesions, prolonged healing time, HSV shedding into the saliva, and the involvement of atypical locations [13]. Diagnosis of HSV infection typically includes clinical examination, Tzanck test, electron microscopy, viral culture, and polymerase chain reaction (PCR) detection of HSV DNA that can be used for confirmation [16,17]. In this case, a biopsy was performed due to the atypical presentation of the lesions, with histopathology subsequently confirming the diagnosis of infectious dermatitis consistent with herpes virus infection.

## Conclusions

Increased clinical awareness regarding the atypical presentation of herpes simplex infection is important for physicians treating immunocompromised patients. In the atypical presentation of HSV, the lesions may be generalized, symptomatic, and present with bilateral involvement. HSV infection should be included in the differential diagnosis along with other vesiculobullous diseases.
